# Short-term effects of video-based education on occupational safety knowledge among commercial divers

**DOI:** 10.3389/fpubh.2026.1799866

**Published:** 2026-04-15

**Authors:** Hideharu Nishikiori, Tatsuya Ishitake

**Affiliations:** 1 Department of Environmental Medicine, Division of Social Medicine, Graduate School of Medical Sciences, Kurume University Kurume, Kurume, Japan; 2 Department of Environmental Medicine, Kurume University School of Medicine, Kurume, Japan

**Keywords:** behavioral change, commercial diver safety, commercial divers, continuing professional development, educational video intervention, occupational diving, occupational safety and health, safety knowledge improvement

## Abstract

**Background and objectives:**

Video-based learning has improved educational outcomes in medicine and nursing. However, in the field of commercial diving, despite the broad range of knowledge required, the effectiveness of license renewal and continuing education systems has not been sufficiently examined. This study evaluated the short-term effects of a video-based educational intervention on OSH knowledge and learning motivation among commercial divers and examined its usefulness as a CE tool.

**Methods:**

A web-based quasi-experimental single-group pre–post intervention study was conducted between November 2024 and August 2025 with the cooperation of the Japan Dive Association. The target population comprised approximately 3,000 commercial divers from 160 corporate member companies. The intervention included a baseline questionnaire, a 13-item OSH knowledge test, an educational video, a post-test, and a final questionnaire. Eighty-one valid responses were analyzed using a paired t-test, with significance set at *p* < 0.05.

**Results:**

The mean correct response rate increased significantly from 46.6 to 66.3% after the intervention (*p* < 0.05). The mean number of correct answers increased from 6.1 to 8.6 (*p* < 0.0001). Knowledge improved for decompression sickness, high-altitude diving, and arterial gas embolism, whereas improvement was limited for M-values in decompression theory and pulmonary oxygen toxicity. Participants also reported increased motivation for information seeking and digital learning.

**Conclusion:**

Education using short answer-explanation videos improved short-term knowledge of the key topics examined and enhanced learning motivation among commercial divers. This approach may support the development of continuing education (CE) programs.

## 1 Introduction

High-skill and knowledge-intensive fields such as medicine and nursing have increasingly reported the effectiveness of video-based educational methods in recent years. For example, in clinical medical education, case-based learning using interactive videos has been shown to improve students’ understanding and learning outcomes (1). In surgical skills education, video-based learning has also been reported to be effective for the acquisition of basic operative skills and may promote the development of knowledge and technical competence compared with conventional teaching methods (2). Thus, video materials accompanied by visual information have attracted attention as an educational approach that facilitates understanding of specialized skills and contributes to improved knowledge retention and memory. In the recreational diving as well, much of the theoretical and safety education is delivered in video-based formats, and video learning is increasingly being adopted as a common educational method. In Japan, the maintenance and repair of infrastructure constructed during the period of rapid economic growth, as well as countermeasures and recovery efforts for natural disasters such as large-scale earthquakes, have become urgent issues. In response, the government is promoting comprehensive disaster prevention and mitigation measures, strengthening port functions to maximize stock effects, and advancing regional revitalization that contributes to Regional Revitalization 2.0 (3). Within this context, commercial divers in Japan are engaged in underwater work in marine environments, including oceans, dams, and rivers. Although the number of occupational accidents associated with diving operations had been decreasing over the years, this trend has recently plateaued. According to Japanese nationwide survey, previous questionnaire surveys on the safety of occupational diving have indicated that the educational content provided to commercial divers has been insufficient (4). Accordingly, in 2020, we conducted an anonymous nationwide questionnaire survey in Japan among active commercial divers in Japan, focusing on their current awareness of occupational safety and health, continuing education, diving conditions, and lifestyle habits (5). The results suggested that improving safety awareness among commercial divers may lead to a reduction in occupational accidents during diving operations. However, the national commercial diver qualification, which is based on the Ordinance on Safety and Health of Work under High Pressure (6), does not mandate periodic renewal or continuing education after certification. As a result, opportunities for education that could contribute to improving occupational safety and health awareness remain limited. Consequently, individual differences in educational exposure and safety awareness are likely to arise, and the updating of safety-related information may be insufficient.

To date, most studies targeting commercial divers have focused on analyses of accident cases, reports on medical symptoms, and cross-sectional surveys of safety behaviors (7, 8). Studies that have examined knowledge acquisition or behavioral change through educational interventions are extremely limited. In particular, with regard to research focusing on the relationship between understanding decompression theory and decompression procedures and safe behavior, a knowledge-level assessment study conducted by Karadurmus et al. among commercial divers in Turkey reported substantial disparities in knowledge according to certification level, occupation, and years of experience (9). However, that study did not evaluate the effects of any intervention, and to date, few studies have examined whether structured educational programs improve knowledge or change safety awareness.

Based on these international and institutional gaps, the present study aims to administer a questionnaire on occupational safety and health, a knowledge assessment test, and video-based educational content covering key occupational safety and health topics to commercial divers, followed by a post-intervention knowledge assessment test, in order to examine whether this intervention approach enhances safety awareness among participants. In addition, this study aims to evaluate the short-term effects of educational videos and knowledge assessment tests as an intervention, to verify their utility for the development of a stepwise educational program, and to explore effective educational tools and methods for providing continuing education opportunities related to occupational safety and health for commercial divers.

## 2 Methods

In our previous study (5), we identified the importance of the following four elements for future occupational safety education for commercial divers.

- Introduction of a renewal system: provision of continuous learning opportunities through e-learning and similar platforms, along with regular updates of essential fundamental knowledge.
- Dissemination of standardized decompression tables: utilization of these tables within practical, work-relevant training.
- Reduction of regional disparities in recompression treatment systems: improvement of regional access to medical care through collaboration with local governments.
- Strengthening of emergency response capacity: expansion of practical education through hands-on training and simulations.

A new questionnaire on occupational safety and health, a knowledge confirmation test, and an educational video explaining key occupational safety and health topics were developed based on these findings. Research cooperation was requested from the Japan Dive Association, and upon obtaining its approval, study invitations were sent by post and email to all 160 corporate members engaged in commercial diving operations. The invitation included information about the questionnaire survey, the knowledge confirmation test, the educational video, and the URL and QR code for web-based access. Commercial divers who received the URL or QR code first read the explanatory document online, provided informed consent electronically, and then proceeded to the baseline questionnaire. They subsequently completed the knowledge confirmation test and, after finishing the test, viewed the educational video. Following the video, they completed the knowledge confirmation test again and then responded to the final questionnaire. The survey period was from November 2024 to August 2025.

### 2.1 Study design

This study was a web-based, quasi-experimental, single-group pre–post intervention study without a control group, designed to evaluate the short-term effects of an educational intervention using video materials for commercial divers. All participants were assessed for changes in occupational safety and health (OSH) knowledge and learning motivation before and after the intervention. Outcomes were measured using a structured questionnaire and a 13-item knowledge confirmation test.

### 2.2 Participants

The target population of this study comprised commercial divers employed by all corporate member diving companies affiliated with the Japan Dive Association (160 companies; estimated total of approximately 3,000 divers). During the study period, study information was distributed to all corporate members through the Association, thereby enabling their affiliated commercial divers to access the study website. Accordingly, this study was conducted as a census of all commercial divers employed by companies affiliated with the Association.

### 2.3 Measures

The questionnaire consisted of 45 items covering the working environment, safety and health behaviors, health effects, educational history, knowledge and understanding, lifestyle habits, and post-intervention changes in awareness. The 13-item knowledge confirmation test was developed using a Japanese government-issued textbook for the national commercial diver examination and questions that had appeared in past examinations. The textbook consists of four chapters: (1) diving operations, (2) air supply, descent, and ascent, (3) hyperbaric disorders (diving-related disorders), and (4) relevant laws and regulations. Of the 13 questions, four (items 5, 6, 7, and 10) addressed topics specific to Japanese laws and regulations, four (items 1, 2, 3, and 4) concerned decompression theory, including descent and ascent, and five (items 8, 9, 11, 12, and 13) concerned hyperbaric disorders. Thus, the questions were distributed across topics other than general diving operations. The number of correct answers and the correct response rate were evaluated according to participant characteristics based on responses to the test administered before and after the educational video intervention.

### 2.4 Intervention

Table 1 presents the 13 items used in the knowledge confirmation test, their main content, and the playback time for each segment; the total video length was approximately 17 min. The educational video consisted of answer explanations corresponding to all 13 test items. With regard to the language of the video, because the Japanese commercial diver examination is conducted only in Japanese, the educational video was also produced in Japanese. The delivery platform was created using Google Forms, in which the questionnaire and tests were arranged. The educational video was provided via a URL linked within the Google Form. Regarding viewing conditions, the platform was designed to be accessible on personal computers, tablets, and smartphones. Most of the video screen consisted of explanatory text in Japanese, and no subtitles were provided. To improve compatibility with mobile devices, where text and animations may appear smaller and more difficult to see, professional audio narration was added. With regard to viewing frequency, the video could be viewed only once during the intervention; however, after completion of the intervention study, a separate URL allowing unlimited viewing was provided, together with the test answers. The full text of the 13 questions and the list of correct answers are shown in Supplementary Table 1. No additional topics had been included at this stage. In the final questionnaire, 22 potential topics were presented, and participants were asked to provide their opinions regarding items they would like to see added in the future.

**Table 1:** Educational video content.

Item	Contents	Duration (s)
1. Half-saturated tissue	Tissue half-time theory Six–half-time saturation	37
2. Inert gas partial pressure	Inert gasses used in diving Inert gas partial pressure at atmospheric pressure Unit of inert gas partial pressure Inert gas partial pressure in gas mixtures	93
3. M-value	Concept of M-values	63
4. Buhlmann ZH-L16 model	Basics of decompression calculation Coefficient in the calculation formula	60
5. Pulmonary oxygen toxicity	Pulmonary oxygen toxicity Numerical limits in Japan	47
6. Gas partial pressure limits (Japan)	Gas partial pressure limits for diving in Japan Pressure units and calculation methods Pressure limits and water depth Relevance to Japanese law Oxygen partial pressure during decompression	253
7. M-value safety factor (Japan)	Safety factor and safety value	53
8. Pre-flight surface interval	Changes in guidelines and waiting times Current preflight waiting time	94
9. Altitude diving	National Oceanic and Atmospheric Administration Diving Manual Atmospheric pressure as a function of altitude	72
10. Decompression table (Japan)	Abolition of decompression tables in Japan Japan Dive Association–recommended decompression tables	34
11. Decompression illness (DCI)	Concept of decompression illness	48
12. Arterial gas embolism (AGE)	Pathophysiology and definition of arterial gas embolism Differences from decompression sickness	44
13. Decompression sickness (DCS)	Pathophysiology and definition of decompression sickness Types of decompression sickness	79

This table presents the test questions used in the intervention, the content of the educational video, and the corresponding playback duration. The 13 questions and their explanatory contents are listed sequentially. The topics include elements specific to the Japanese commercial diver licensing system, such as the M-value safety factor and gas partial pressure limits, as well as general diving-related concepts, including decompression theory, oxygen toxicity, inert gas partial pressure, and tissue half-time theory.

### 2.5 Data collection

The data collected through Google Forms were aggregated in a spreadsheet and subsequently converted into an Excel datasheet. This process minimized the risk of data loss and transcription errors during data handling and conversion.

### 2.6 Data analysis

Of the 267 responses that accessed Google Forms, 81 participants were included in the final analysis, as shown in Supplementary Figure 1, because they met all of the following criteria: (1) provided consent to participate in the study, (2) completed the baseline questionnaire, (3) completed the pre-test, (4) completed viewing of the educational video, (5) completed the confirmation test, and (6) completed the final questionnaire. First, bivariate analyses were conducted to examine the associations between participant characteristics and diving work characteristics and the results of the pre-test and post-test before and after viewing the educational video. In addition, years of diving experience (an ordinal variable with five categories) and the timing of commercial diver qualification acquisition were entered as independent variables, and their associations with the number of correct answers were examined using multivariable regression analysis. To evaluate changes in knowledge before and after viewing the educational video, the mean correct response rate and the number of correct answers were calculated for the 13 items in the pre-test and post-test, and comparisons were made using a paired t-test. Statistical analyses were performed using JMP Student Edition 18, and the level of statistical significance was set at 5% (two-sided).

### 2.7 Ethical considerations

This study was conducted with the approval of the Kurume University Ethics Review Committee (Approval no. 24164).

## 3 Results

### 3.1 Basic characteristics and work practices of participants

Table 2 presents the basic characteristics and work practices of the participants. The largest age group was individuals in their 50s (25.9%), followed by those in their 40s and those younger than 30 years (23.5% each). When combined with participants in their 30s (18.5%), individuals younger than 60 years accounted for 91.4% of the sample. Regarding sex, 91.4% of participants were male, 7.4% were female, and 1.2% preferred not to disclose their sex. With respect to body mass index (BMI), the majority of participants were classified as having normal weight (18.5 ≤ BMI < 25.0) (64.2%), followed by those who were overweight (BMI ≥ 25.0) (33.3%). Only 2.5% were classified as underweight (BMI < 18.5). In terms of educational background (highest level attained), 67.9% had graduated from high school or vocational school, 22.5% had graduated from university or graduate school, and 8.6% had graduated from junior high school. Regarding diving experience, participants with less than 10 years of experience constituted the largest proportion (39.5%), followed by those with 10–19 years (17.3%), 20–29 years (19.8%), and 30–39 years (19.8%). The average number of diving days per year was less than 90 days for 37.0% of participants, followed by 90 to fewer than 180 days for 28.4%, and 180 to fewer than 270 days for 34.6%. Regarding the content of diving operations, various survey-related tasks were the most common (39.5%), followed by general civil engineering work excluding stone-laying and wave-dissipating block installation (29.6%), and stone-laying and wave-dissipating block construction (14.8%), together accounting for 83.9% of all activities. Other types of work included fisheries (7.4%), photography and video recording (3.7%), salvage operations (2.5%), and both shipbuilding and rescue operations (1.2% each). Regarding diving methods, the hookah system was the most commonly used, reported by 56.8% of participants. With respect to the timing of obtaining the national commercial diver license, 63.0% of participants had obtained their license before October 2016, when a major revision of the Ordinance on Safety and Health of Work under High Pressure was implemented.

**Table 2:** Occupational health and safety status of study participants.

Item	Number of people	(%)
Age(years), Average(SD)	42.1 (13.1)	
<30 years	19	(23.5)
30–39 years	15	(18.5)
40–49 years	19	(23.5)
50–59 years	21	(25.9)
≧60 years	7	(8.6)
Sex		
Male	74	(91.4)
Female	6	(7.4)
Prefer not to disclose	1	(1.2)
BMI (%), Average(SD)	24.1 (3.9)	
Underweight (BMI < 18.5)	2	(2.5)
Normal weight (18.5≦BMI < 25.0)	52	(64.2)
Overweight (25.0≦BMI)	27	(33.3)
Educational background (highest level completed)		
Junior high school	7	(8.6)
High school	38	(46.9)
Vocational school	17	(21.0)
University, Graduate school	19	(22.5)
Years of diving experience, Average(SD)	16.3 (13.0)	
<10 years	32	(39.5)
10–19 years	14	(17.3)
20–29 years	16	(19.8)
30–39 years	16	(19.8)
≧40 years	3	(3.8)
Annual diving days, Average(SD)	117.2 (79.6)	
<90 days	30	(37)
90–179 days	23	(28.4)
≧180 days	28	(34.6)
Type of diving work		
Various surveys	32	(39.5)
Underwater construction works	24	(29.6)
Block and rock placement works	12	(14.8)
Fishery	6	(7.4)
Filming/video recording	3	(3.7)
Salvage	2	(2.5)
Others (shipbuilding, rescue, etc.)	2	(2.5)
Diving method		
Surface-supplied (e.g., hookah system)	46	(56.8)
Self-contained (e.g., scuba tank)	35	(43.2)
Date of diver’s license acquisition		
October 2016 or earlier	51	(63)
November 2016 or later	30	(37)

This table summarizes the demographic characteristics, educational background, diving experience, annual diving activity, type of diving work, diving method, and timing of diver’s license acquisition among the study participants. (*n* = 81). Data are presented as the number of participants (%) or as the mean ± standard deviation, as appropriate.

### 3.2 Safety and health practices and information-seeking behavior

**Figure 1:**
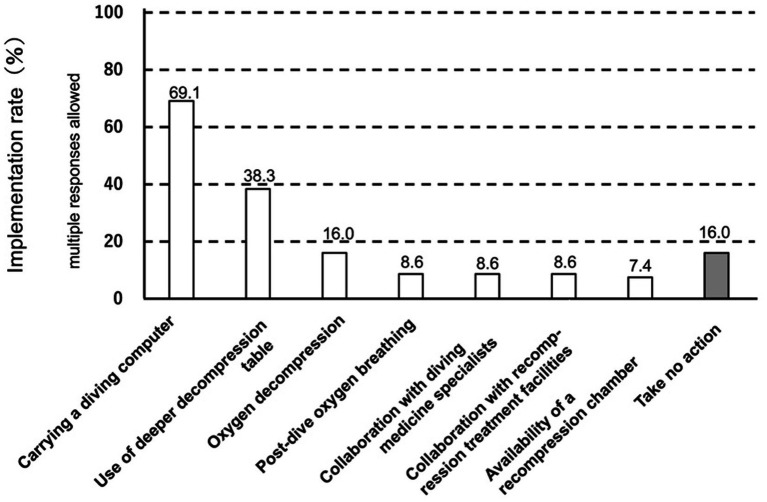
Safety measures commonly implemented by professional divers. The figure shows the implementation rates (%) of safety measures reported by professional divers (*n* = 81), including carrying a diving computer, use of deeper decompression tables, oxygen decompression, post-dive oxygen breathing, collaboration with diving medicine specialists, collaboration with recompression treatment facilities, availability of a recompression chamber, and cases in which no specific safety measures were taken.

Figure 1 shows the safety measures that commercial divers routinely implement (multiple responses allowed). Carrying a diving computer was the most frequently reported measure (69.1%), followed by “depth rank-up,” defined as the use of decompression tables corresponding to a deeper depth category than the actual dive depth (38.3%). Oxygen decompression was reported by 16.0% of participants; however, an equal proportion (16.0%) reported taking no specific safety measures. In contrast, the proportions reporting “post-dive oxygen breathing,” “collaboration with medical specialists,” “coordination with recompression treatment facilities,” and “installation of recompression chambers” were all below 10%.

Although not shown in the figure, responses regarding consultation sources in the event of feeling unwell after diving (multiple responses allowed) indicated that the “company or dive supervisor” was the most common source (55.6%), followed by “senior colleagues or acquaintances” (42.0%) and “physicians specializing in diving medicine” (35.8%). Regarding prior participation in diving-related lectures or study sessions, participation in fewer than five sessions was the most common response (44.4%), followed by no prior participation (38.3%), while participation in five or more sessions was reported by 17.3% of participants. Concerning motivation to seek safety-related information, “general motivation” was most frequently reported (40.7%), followed by “high motivation” (30.9%) and “some motivation if information is provided” (21.0%). With respect to video-based content related to diving safety information, 69.1% of participants indicated that they would like to view such content, 24.7% reported that they were unsure, and 6.2% indicated that they would not view it.

### 3.3 Health-related outcomes

Regarding experience with recompression therapy, 91.4% of participants reported no prior experience. Among those who had undergone recompression therapy, 3.7% reported one experience, 1.2% reported two or three experiences, and 2.5% reported five or more experiences. With respect to perceived treatment outcomes among those with experience, “improved” was the most frequently reported response (57.1%), followed by “persistent sequelae” (28.6%) and “residual discomfort” (14.3%). Regarding physical condition after completion of diving and surfacing, 81.5% of participants reported no post-dive physical symptoms, whereas 18.5% reported experiencing such symptoms occasionally.

### 3.4 Changes in knowledge before and after viewing the educational video

**Figure 2:**
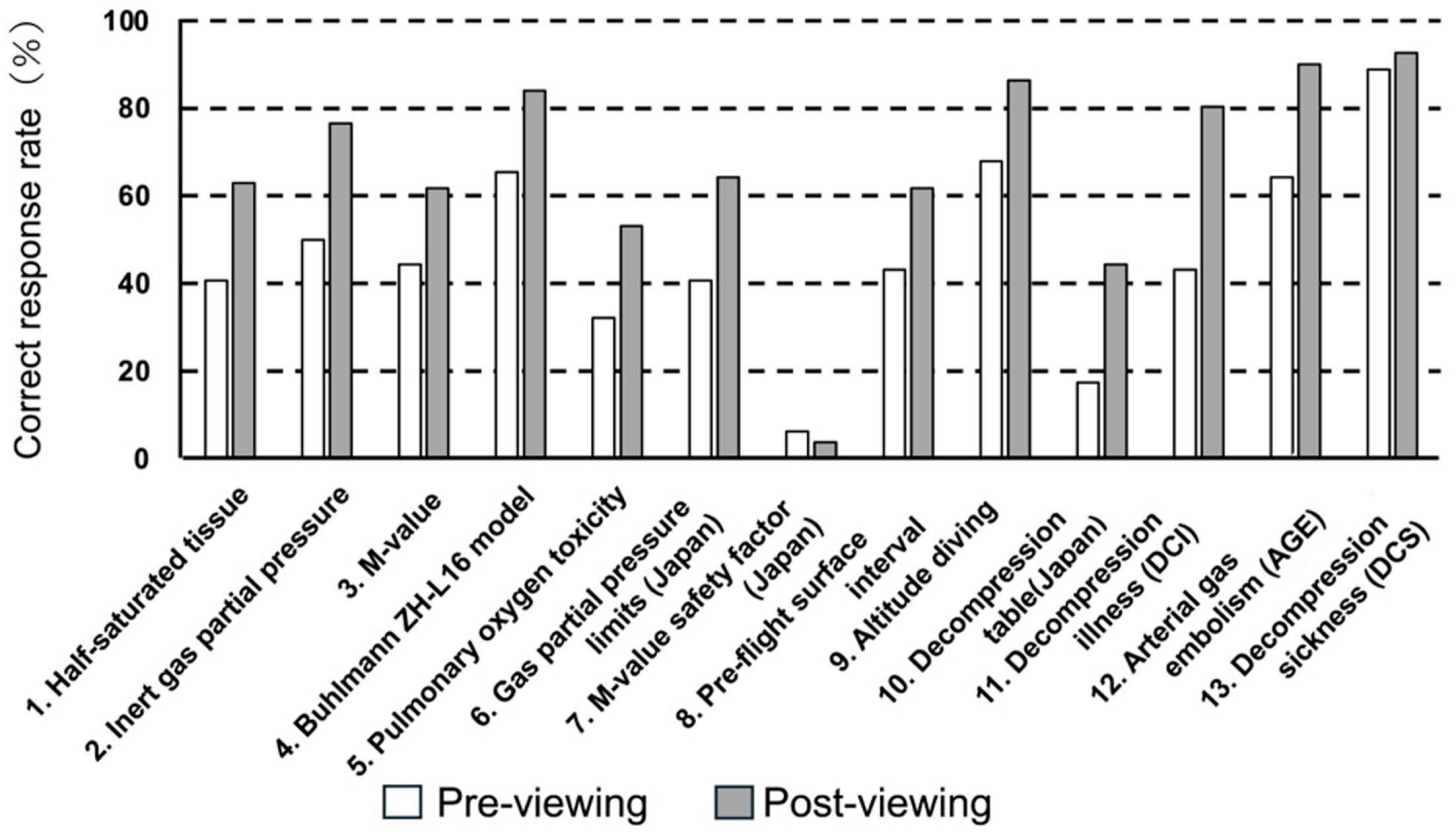
Changes in professional divers’ understanding of specialized knowledge before and after watching the educational video. Correct response rates (%) for 13 domains of diving physiology and safety are shown before and after viewing the educational video (n = 81). The evaluated items include: (1) half-saturated tissue, (2) inert gas partial pressure, (3) M-value, (4) Buhlmann ZH-L16 model, (5) pulmonary oxygen toxicity, (6) gas partial pressure limits in Japan, (7) M-value safety factor in Japan, (8) pre-flight surface interval, (9) altitude diving, (10) decompression tables, (11) decompression sickness, (12) arterial gas embolism, and (13) decompression illness. Open bars indicate pre-viewing, and filled bars indicate post-viewing.

Figure 2 illustrates changes in knowledge levels before and after viewing the explanatory educational video. The mean correct answer rate on the pre-intervention test across the 13 questions was 46.6%. Following the educational video, the mean correct answer rate on the post-intervention knowledge assessment test increased to 66.3%. The mean number of correct answers increased from 6.1 before the intervention to 8.6 after the intervention, representing a statistically significant improvement (*p* < 0.0001, paired t-test). On the pre-intervention test, the highest correct answer rate was observed for “decompression sickness” (88.8%), followed by “high-altitude diving” (67.9%), the “Buhlmann ZHL model” (65.4%), and “arterial gas embolism” (65.4%). These were the only four items with correct answer rates exceeding 60% prior to the intervention. In contrast, the lowest pre-intervention correct answer rate was observed for “the safety margin of the M-value in the Ordinance on Safety and Health of Work under High Pressure” (6.2%), followed by “decompression tables under the Ordinance on Safety and Health of Work under High Pressure” (17.3%) and “pulmonary oxygen toxicity” (32.1%). Comparisons before and after viewing the educational video showed increases in correct answer rates for all items except “the safety margin of the M-value in the Ordinance on Safety and Health of Work under High Pressure.” However, even after the intervention, correct answer rates did not reach 60% for three items: “pulmonary oxygen toxicity,” “the safety margin of the M-value in the Ordinance on Safety and Health of Work under High Pressure,” and “decompression tables under the Ordinance on Safety and Health of Work under High Pressure”.

Although these data are not presented, no significant associations were observed between the correct answer rate and either the timing of commercial diver license acquisition or the number of diving days per year. In addition, in bivariate analyses and multiple regression analyses using the pre-intervention number of correct answers as the dependent variable, no independent variables showed statistically significant associations. Furthermore, when years of diving experience (categorized into five groups) were analyzed as an ordinal variable, no significant association was found with the pre-intervention number of correct answers. The timing of commercial diver license acquisition was also compared between those who obtained the license before and after the 2016 revision of the examination questions; however, no statistically significant difference in correct answer rates was observed between the groups. Moreover, neither the number of diving days per year nor other experience-related variables showed significant associations in either univariate or multivariate analyses.

### 3.5 Knowledge assessment test, educational video content, and changes in awareness before and after the intervention

**Figure 3:**
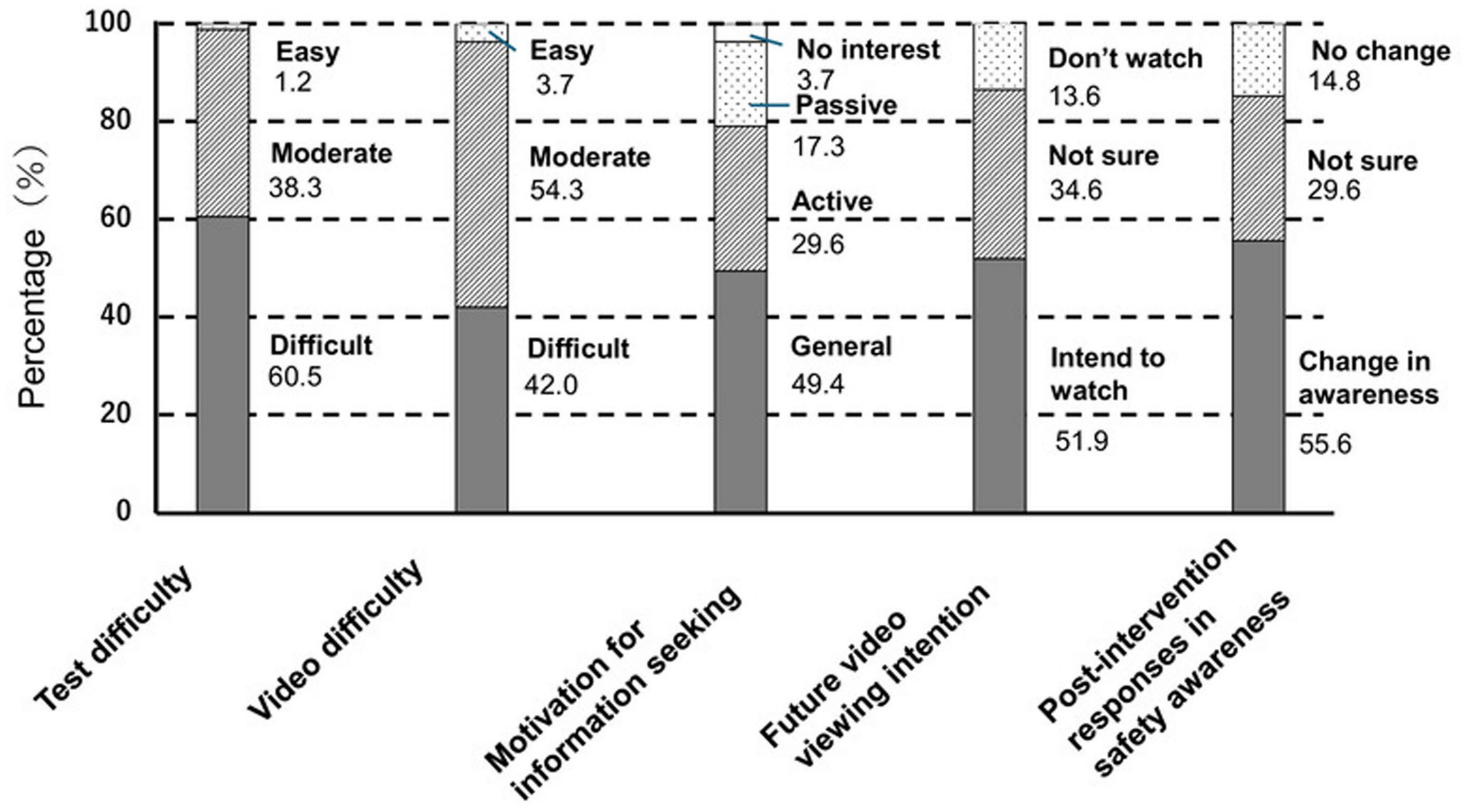
Changes in awareness and attitudes after viewing the educational video. The proportions (%) of participants reporting changes in perceived test difficulty, video difficulty, motivation for information seeking, intention to view future educational videos, and safety awareness after watching the educational video are presented (*n* = 81). Percentages may not total 100% because of rounding.

Figure 3 presents changes in awareness following viewing of the educational video. Regarding the knowledge assessment test, 60.5% of participants reported that the content was “difficult,” 38.3% reported that it was “moderate,” and only 1.2% reported that it was “easy.” With respect to the content of the explanatory educational video, 42.0% of participants reported that it was “difficult,” while the largest proportion reported that it was “moderate” (54.3%), and 3.7% reported that it was “easy.” Regarding changes in awareness after viewing the video, motivation to seek safety-related information was most commonly reported as “general motivation” (49.4%), followed by “high motivation” (29.6%) and “some motivation if information is provided” (17.3%); 3.7% reported no interest. When asked whether they would like to view similar video content in the future, the most common response was “would like to continue viewing” (51.9%), followed by “unsure” (34.6%), while 13.6% reported that they would not view such content. With respect to perceived changes in awareness following the intervention, 55.6% of participants reported that their awareness had changed, 29.6% reported that they were unsure whether it had changed, and 14.8% reported that it had not changed.

**Figure 4:**
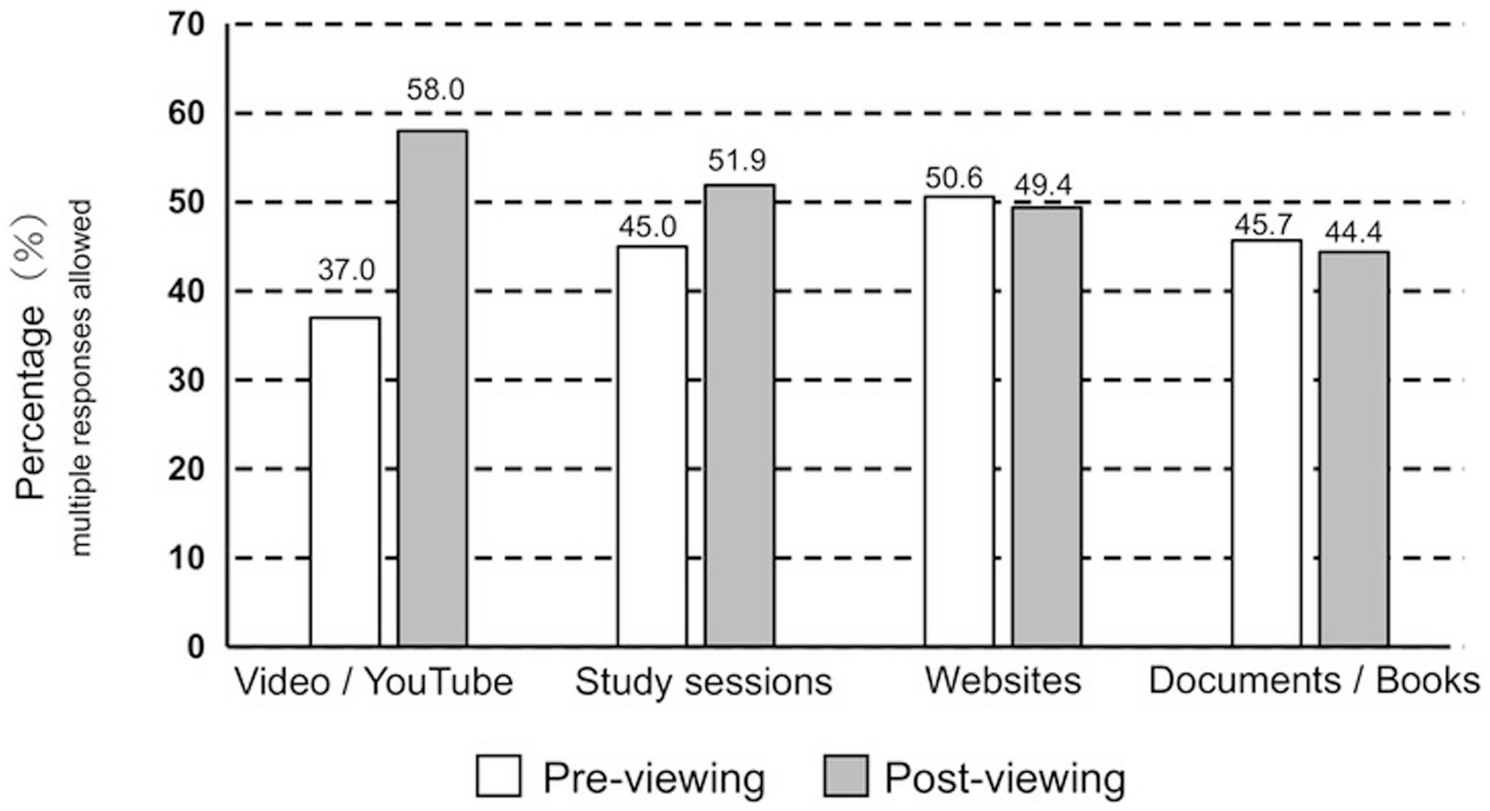
Changes in information-gathering methods before and after watching the video. The proportions (%) of information sources used by professional divers before and after video viewing are shown (*n* = 81), including video platforms (e.g., YouTube), study sessions, websites, and documents or books. Pre-viewing and post-viewing conditions are compared.

Figure 4 illustrates changes in information-seeking methods (multiple responses allowed) before and after viewing the educational video. Before viewing the video, the most frequently reported information source was “websites (internet)” (50.6%), followed by “books and printed materials” (45.7%), “study meetings or seminars” (45.0%), and “videos or YouTube” (37.0%). After viewing the video, a shift was observed, with “videos or YouTube” becoming the most frequently selected source (58.0%), followed by “study meetings or seminars” (51.9%), “websites (internet)” (49.4%), and “books and printed materials” (44.4%). These results indicate that information sources selected after the intervention were not exclusively digital, with analog sources remaining similarly utilized.

## 4 Discussion

### 4.1 Current status and challenges of safety and health practices and awareness

This study clarified the current status of safety and health practices and awareness among commercial divers and demonstrated that an educational intervention can improve occupational safety and health–related knowledge as well as motivation toward continuing education. Internationally, a study targeting commercial divers in Turkey reported substantial variability in knowledge related to decompression theory and safety procedures depending on certification type and work domain, particularly in aquaculture and individual diving operations (9). However, in contrast to the findings from Turkey, the present results indicate that among Japanese commercial divers, knowledge levels were not dependent on individual factors such as years of experience or specific work domain. This difference is likely attributable to variations in national qualification systems, renewal requirements, and initial training structures between countries, as discussed below, suggesting that the need for educational interventions is a common international challenge.

With respect to continuing education, a large proportion of participants expressed a desire to collect information related to diving safety, regardless of whether the format was digital or analog. Moreover, the mean correct answer rate prior to viewing the educational video was 46.6%, which was below 50% and lower than the level initially anticipated by the authors. This finding suggests that fundamental knowledge may not be sufficiently retained among commercial divers as a whole. In particular, items requiring theoretical understanding—such as the “safety margin of the M-value under the Ordinance on Safety and Health of Work under High Pressure” and the “decompression tables under the same ordinance”—remained below a 60% correct answer rate even after viewing the educational video. This indicates that, although actual diving operations may be carried out based on accumulated experience and customary practices, accurate foundational knowledge of decompression theory and high-pressure environments may not be systematically or formally acquired. Such a gap between practice and knowledge may constitute a structural factor that allows risks such as decompression illness to be overlooked. Therefore, reliance on on-site experience alone is insufficient, and there is a clear need for the continuous provision of scientifically grounded educational content, along with mechanisms to visualize learning outcomes. Establishing such educational systems is likely to contribute to the development of a strong safety culture and to the prevention of occupational accidents. On the other hand, participation in diving-related lectures and study meetings was found to be limited. This may be attributable to the absence of a renewal system for the commercial diver license itself, the limited number of available lectures and study meetings, or geographic disparities in their availability. As a result, it is likely that commercial divers face substantial barriers to independently seeking out and attending educational opportunities.

Many participants reported “depth rank-up” as a safety measure; however, this practice is closely related to the safety margin applied to the M-value, defined as the maximum allowable level of inert gas in tissues that is considered not to result in decompression illness. Despite this, the pre-intervention test showed an extremely low correct answer rate regarding the concept of safety margins. Although this issue is not explicitly stipulated in the Ordinance on Safety and Health for Work under High Pressure, the related Notification No. Ki-hatsu 0109–2, issued in 2015 by the Director-General of the Labor Standards Bureau, Ministry of Health, Labor and Welfare (Japan), merely states that “a high safety margin should be adopted when calculating M-values” (10) and provides no explanation of how such safety margins should be calculated. This lack of methodological guidance is presumed to be a major factor contributing to the considerable difficulty in understanding and applying M-values in practice.

Post-intervention areas of interest (multiple responses allowed) included “descent and ascent procedures,” which was the most frequently selected topic (37.0%), followed by “laws and regulations related to diving” (28.4%), “consultation services for decompression-related disorders” (25.9%), and “nitrogen narcosis” (24.7%). “Nationwide emergency recompression facilities,” “prohibited work practices,” and “oxygen decompression” were each selected by approximately 23.5% of participants. In addition, regarding confirmation of dive planning, the proportion of participants who reported “myself” as the primary responsible person increased from 45.7 to 51.9%, whereas the proportion reporting “the client or prime contractor” decreased from 29.6 to 22.2%. In addition, responses regarding video content of interest indicated demand for information on consultation services for decompression-related disorders and nationwide emergency recompression facilities. This suggests that issues related to detailed planning for diving accidents and transportation challenges arising from the uneven geographic distribution of emergency recompression facilities remain unresolved. However, because most individual topics of interest were selected by fewer than 30% of participants, further efforts are needed to increase engagement and interest. Finally, as approximately half of the participants indicated that dive planning should be confirmed by the divers themselves, these findings suggest a clear need for detailed dive planning and emergency response planning in the event of diving accidents.

### 4.2 Learning effects of the educational video intervention

In the present intervention, the mean number of correct answers across the 13 items increased significantly from 6.1 before viewing the educational video to 8.6 after viewing (*p* < 0.0001), demonstrating a short-term educational effect in terms of knowledge acquisition through the video-based intervention. The evaluation conducted in this study was based on a knowledge assessment test administered immediately after the intervention and can therefore be positioned as capturing a short-term outcome corresponding to the “learning” level in the educational evaluation model proposed by Kirkpatrick et al. (11).

Importantly, the knowledge improvement observed in this study reflects a short-term effect only, and it was not possible to assess whether the acquired knowledge was retained over time. Previous studies have shown that, in educational interventions in medical and safety-related fields, significant improvements in knowledge and skills are often observed immediately after the intervention, whereas declines in knowledge and skills tend to occur at follow-up several months later. For example, Odongkara et al. reported such patterns of short-term improvement followed by subsequent decline (12). Similarly, studies of video-based educational interventions by Saidu et al. observed attenuation of knowledge and skills at follow-up evaluations conducted 3–6 months after the intervention (13). Robson et al. also reported significant short-term improvements in knowledge following educational interventions and emphasized the importance of long-term knowledge retention (14). In the fields of educational psychology and the learning sciences, it has likewise been demonstrated that single-session learning interventions can be effective in the short term but have inherent limitations with respect to long-term knowledge retention. Roediger et al. proposed that the act of testing itself promotes long-term memory consolidation through what is known as the testing effect (15). Cepeda et al. further demonstrated that spaced repetition, or distributed learning with intervals between learning sessions, is effective for long-term knowledge retention, and that even repeated testing using the same questions after viewing educational videos can contribute to sustained learning (16). These prior findings theoretically support the necessity of incorporating periodic review (repeated learning) and testing-effect–based educational interventions into occupational safety and health education for commercial divers.

Although this study focused on evaluating the short-term effects of an educational intervention, assessment of short-term outcomes does not necessarily represent an insufficient evaluation. In the fields of learning science and occupational safety education, the validity of evaluating short-term changes such as knowledge acquisition and cognitive understanding as primary outcomes of educational interventions is widely accepted. Moreover, short-term improvements in knowledge should not be regarded as merely transient phenomena, but rather as an essential foundation for subsequent knowledge retention and behavioral change. From this perspective, the present findings provide important baseline evidence to support future long-term evaluation studies.

### 4.3 Comparison with international diving education programs

When Japan’s diving education system is compared with overseas diving education systems and mutual recognition frameworks, it becomes evident that, internationally, many countries and organizations—including the International Diving Schools Association (IDSA) (17), the International Marine Contractors Association (IMCA) (18), the Association of Diving Contractors International (ADCI) (19), the Health and Safety Executive (HSE) in the United Kingdom (20), and Safe Work Australia (21)—have legally adopted frameworks for occupational diving education and safety management. These frameworks ensure educational compliance, mutual recognition, and acceptance within the industry, and they institutionally guarantee the maintenance of qualifications and the continuation of safety education. In the United Kingdom, the Health and Safety Executive (HSE), and in Norway, the Norwegian Ocean Industry Authority (Havtil) (22), as well as relevant authorities in other countries, mutually recognize IDSA standards (Mutual Recognition) (23), while issuing national diving licenses in accordance with domestic legislation.

Requirements for renewal and requalification vary by country; however, in the United States under ADCI, in Australia under the Australian Diver Accreditation Scheme (ADAS) (24), and in Canada under the Diver Certification Board of Canada (DCBC), the validity period of certification is generally set at 5 years (with a two-year validity period for initial certification only in Canada) (25), making periodic renewal mandatory.

Regarding the use of e-learning, some IDSA member schools have digitized foundational education in diving theory and safety management. In contrast, IMCA’s e-learning programs are primarily operated as continuing professional development (CPD) initiatives for already certified divers and supervisors, focusing on safe work practices and international standards such as the IMCA D-series.

Among countries that operate independent systems under domestic law, Turkey (26), like Japan, represents a unique case; however, its educational content and structure differ substantially from those of Japan. In Turkey, completion of a two-year specialized diving course, including both theoretical and practical components, is a prerequisite for employment as a commercial diver. Similar to systems in Europe and North America, Turkey has a structured framework in which higher-level qualifications are obtained through the accumulation of education and work experience.

In contrast to these international systems, Japan’s current framework allows certification based solely on a written examination without a practical skills assessment and lacks both a license renewal system and a structured mechanism for periodic education. As a result, Japan’s domestically confined commercial diver certification system, which is not linked to international frameworks, has structural limitations that make it vulnerable to regional disparities and unequal access to educational opportunities. Furthermore, Karadurmus et al. (9) reported that, in Turkey, certification category, work domain, and years of experience were significantly associated with knowledge levels. The authors suggested that these differences reflected variation in educational content across qualification categories, including the presence or absence of effective training, more advanced curricula, and continuing education, which in turn contributed to the separation of groups with higher and lower knowledge scores. In other words, differences in the educational system appear to have produced differences in knowledge levels. In contrast, in Japan, neither years of experience nor work domain was associated with pre-intervention test performance. Based on the present findings, both initial education and continuing education appear to be insufficiently developed, and the overall levels of correct answers and correct answer rates suggest that Japanese commercial divers may constitute a relatively homogeneous group without clear stratification according to educational background, largely because of limited formal diving education. Therefore, within the same certification category, years of experience and work domain may not have been associated with knowledge levels. Although the findings from the two countries may appear different at first glance, when interpreted from the perspective of stratification by educational framework, the present results are not contradictory to the Turkish findings but rather broadly consistent with them.

### 4.4 Specific recommendations and feasibility of implementation

Based on the demonstrated effectiveness of the educational intervention for commercial divers in this study, we propose the following recommendations.

- Adoption of globally standardized diving education: Advocate to relevant organizations, academic societies, and governmental bodies for the adoption, alignment, certification, and acceptance of standardized global diving education and renewal systems.
- Development of educational videos: Create clear and accurate educational content that improves correct answer rates while avoiding ambiguity or misunderstanding.
- Promotion of educational video dissemination: Utilize new media and social networking services, such as LINE advertisements and other digital platforms, to promote the use of digital educational content.
- Development of detailed emergency response plans for diving accidents: Provide support for the creation of comprehensive plans covering transportation methods, receiving medical facilities, diving profiles, diver data sheets, and coordination with relevant regulations such as the High Pressure Gas Safety Act.
- Physician support during emergencies: Secure access to physicians specializing in diving medicine who can serve as consultation resources during emergencies, and establish robust backup support systems.
- Introduction of practical skills training: Implement training programs covering essential diving skills, decompression procedures, and emergency response measures.

With regard to feasibility, the ultimate goal would be to incorporate globally standardized education and to adopt, align with, certify, or accept frameworks recognized by organizations such as IDSA or ADCI. However, given the historical context of domestic legal revisions and the development of the commercial diver licensing system in Japan, achieving this goal is not straightforward. Although introducing a license renewal system for commercial divers would be a logical first step, it is not currently realistic under existing domestic conditions. In this context, a more practical approach would be to follow examples from Turkey and East Asian countries, gradually elevating Japan’s educational content to an international level while operating within the framework of domestic law. Such an approach would require strong legal and financial support from the national government. As a preliminary step, it is essential to widely communicate to relevant stakeholders that commercial divers engaged in occupational diving are actively seeking safety-related information and educational opportunities, and to provide the necessary support accordingly. Specifically, educational videos should be developed and made broadly accessible, accompanied by active dissemination of information through targeted advertising and outreach. For effective information provision, participation and support from industry organizations and related academic societies are necessary, while governmental involvement is indispensable for securing financial resources.

At present, the Decompression Illness Countermeasures Committee of the Japanese Society of Hyperbaric and Undersea Medicine is undertaking several initiatives, including (A) the development of diagnostic and treatment guidelines for hyperbaric (diving-related) disorders; (B) the establishment of a response network for hyperbaric (diving-related) disorders; and (C) the creation of a registry for cases of hyperbaric (diving-related) disorders (27). In particular, initiative (B) is closely related to the uneven geographic distribution of Type II hyperbaric treatment facilities and has prompted a review of safety standards for hyperbaric oxygen therapy using Type I chambers. Furthermore, with respect to the development of diving medical plans and physician support during accidents, implementation may be facilitated through outsourcing to consultants in collaboration with specialized institutions. Such arrangements would enable effective support for both diving operators and commercial divers, thereby enhancing the practical feasibility of these recommendations.

### 4.5 Significance and limitations of this study

This study represents the first intervention study to employ educational videos targeting commercial divers and constitutes a pioneering effort to quantitatively evaluate improvements in occupational safety and health–related knowledge and learning motivation. A statistically significant increase in the mean number of correct answers—from 6.1 to 8.6—was observed before and after viewing the educational video, clearly demonstrating a short-term effect on knowledge acquisition. This finding has important academic significance. In addition, more than half of the participants reported increased motivation to seek safety-related information after viewing the video, providing empirical support for the usefulness of digital learning tools in continuing education. These findings are consistent with international trends promoted by organizations such as the International Diving Schools Association (IDSA), the International Marine Contractors Association (IMCA), the Health and Safety Executive (HSE), the Association of Diving Contractors International (ADCI), and Safe Work Australia, all of which emphasize continuing education based on license renewal, competency assessment, and e-learning. Accordingly, the present study offers valuable insights for the development of a systematic continuing education model in Japan.

However, this study has several methodological limitations:

First, the study employed a single-group pre–post design without a control group, which precludes rigorous separation of the effects of the educational intervention from potential confounding factors.
Second, the analytical sample was limited to 81 participants, and this relatively small sample size restricts statistical power.
Third, of the 267 total website accesses, only 81 responses were ultimately valid, indicating an attrition rate of approximately 70%. This high dropout rate may be attributable to the demanding work environment of commercial divers, irregular working hours, constraints related to communication infrastructure, or the possibility that the educational video format was unfamiliar or less acceptable to some participants. Although no analysis of dropout characteristics was conducted, this limitation may affect sample representativeness and the generalizability of the observed educational effects. Future studies should examine reasons for attrition in greater detail.
Fourth, knowledge in this study was defined using a 13-item test; however, the validity of this instrument has not been formally verified. As a result, the extent of measurement error remains unknown, which represents a methodological limitation and constrains the interpretability of the results.
Fifth, the 13-item knowledge test was not designed to comprehensively evaluate the full spectrum of theoretical knowledge required of commercial divers. Instead, the items were selected as core occupational safety and health topics that are repeatedly addressed in the Japanese national examination for commercial divers and are directly relevant to safe diving practices and the prevention of diving-related disorders. Recent national examination questions indicate that the examination encompasses a broader framework consisting of four categories: “diving operations,” “air supply, descent, and ascent,” “hyperbaric disorders,” and “related laws and regulations.” The 13 items used in the present study are clearly included within this broader framework. Nevertheless, they do not cover the full range of knowledge required of commercial divers. Furthermore, education using short videos that explained the answers improved short-term understanding of the key topics and learning motivation; however, because the intervention evaluated effectiveness within the extremely limited scope of only 13 topics, the findings may overestimate the educational effect for commercial divers and should therefore be interpreted with caution. This point represents one of the limitations of the study.
Sixth, the study protocol approved by the ethics review committee adopted a staged consent process, in which participant consent was obtained at each step of the research procedure, including consent to participate, completion of the pre-questionnaire, pre-test, video viewing, and final questionnaire. In accordance with this ethical agreement, data from individuals who did not complete the study and did not provide full consent for the final stage were not eligible for analysis. Therefore, although partial data were technically available for some participants who dropped out during the study, these data could not be analyzed because of the ethical restrictions defined in the approved study protocol. We acknowledge that the inability to examine the characteristics of dropouts is a limitation of the present study.

In the present study, no significant associations were found between correct answer rates and either the timing of commercial diver license acquisition or the number of diving days per year. In addition, neither the bivariate analysis nor the multiple regression analysis, in which the pre-intervention number of correct answers was used as the dependent variable, identified any independent variables showing a statistically significant association. Furthermore, when years of diving experience were analyzed as an ordinal variable with five categories, no significant association was observed with the pre-intervention number of correct answers. The timing of commercial diver license acquisition was also compared between those who obtained the license before and after the 2016 revision of the examination, but no statistically significant differences in correct answer rates were found between the groups. Moreover, no significant associations were identified for annual diving days or other experience-related variables in either univariate or multivariable analyses. These findings suggest that comprehension of the educational video and knowledge acquisition in this intervention may depend more strongly on factors such as opportunities for continuing learning, educational content, and instructional systems than on diving experience itself. Furthermore, the knowledge gains observed in this intervention were limited to short-term effects, and the durability of learning outcomes over time, as well as their translation into actual safety behaviors, remains unclear. With respect to the educational video content itself, correct answer rates for certain items—such as the safety margin of the M-value under the Ordinance on Safety and Health of Work under High Pressure—remained low, indicating that some topics may not be sufficiently understood through a single video-based session. This finding suggests areas for improvement in instructional design, including excessive terminological complexity and insufficient scaffolding to support comprehension. In particular, when addressing regulations or numerical standards that are not adequately explained within the existing legal framework, such as methods for calculating safety margins, it is necessary to present legal rationales and scientific backgrounds in a clearer and more accessible manner.

### 4.6 Future research directions

The present study demonstrated that an educational video–based intervention has a measurable effect on improving knowledge and raising awareness among commercial divers. However, to further develop these findings and institutionalize them as a sustainable framework for safety education, several issues warrant further investigation.

First, international comparative research is needed to comprehensively examine the current status of diver education systems and licensing frameworks across countries. In particular, systematically organizing information on the relationships between training providers and licensing authorities, the degree of standardization of educational content, and the presence or absence of continuing education and recertification systems would provide practical insights for the development of a globally aligned education system in Japan.

Second, qualitative improvement of educational content and strengthening of dissemination systems are required. In the present materials, certain items showed limited comprehension, indicating the need to revise specialized content to ensure clarity, define technical terminology, and provide Supplementary material explanations of legal interpretations to avoid misunderstanding. To ensure accuracy and reliability, it is desirable to establish a multidisciplinary review system involving medical and legal experts and to implement a multi-stage content validation process.

Third, optimization of content dissemination methods and outreach strategies should be examined. This includes combining video-based learning with face-to-face training such as on-site workshops and study meetings, building collaborative dissemination frameworks with industry organizations, vocational training institutions, and diver registration authorities, and developing wide-reaching communication strategies using the internet and social networking services.

Fourth, methodological refinement of intervention studies and expansion of sample size are necessary. In this study, the sample size was limited and the dropout rate was high. Future research should therefore establish larger-scale survey frameworks to collect representative data from divers with diverse employment conditions, ages, and levels of experience. Where feasible, conducting randomized controlled trials with control groups, as well as implementing long-term follow-up studies to evaluate the durability of learning effects and subsequent behavioral change, would enable more rigorous evaluation of educational interventions.

## 5 Conclusion

This study represents the first national investigation to evaluate the effects of an educational video–based intervention for commercial divers and provides empirical evidence of short-term improvements in knowledge and increased learning motivation. The significant increase in mean correct answer rates following video viewing, together with the observed rise in preference for digital learning, highlights the potential of video-based education to expand opportunities for on-site safety training. By developing a stepwise educational program that combines repeated educational videos with knowledge assessment tests over relatively short intervals, it may be possible to promote long-term retention of occupational safety and health knowledge and facilitate behavioral change among commercial divers. Future efforts should focus on (A) refinement of educational content, particularly in areas such as M-values and oxygen toxicity; (B) ensuring representativeness through large-scale surveys; (C) tracking long-term learning outcomes and behavioral change; and (D) integrating educational interventions with license renewal systems and industry-based training programs. Through these strategies, the development of a sustainable safety culture and the reduction of occupational accidents in commercial diving can be advanced.

## Data Availability

The original contributions presented in the study are included in the article/Supplementary material, further inquiries can be directed to the corresponding author/s.
