# Supplementary figures and images for: Short-term effects of video-based education on occupational safety knowledge among commercial divers
Source: Front Public Health. 2026 Apr 15;14:1799866. doi: 10.3389/fpubh.2026.1799866 (PMC13127255; doi:10.3389/fpubh.2026.1799866)

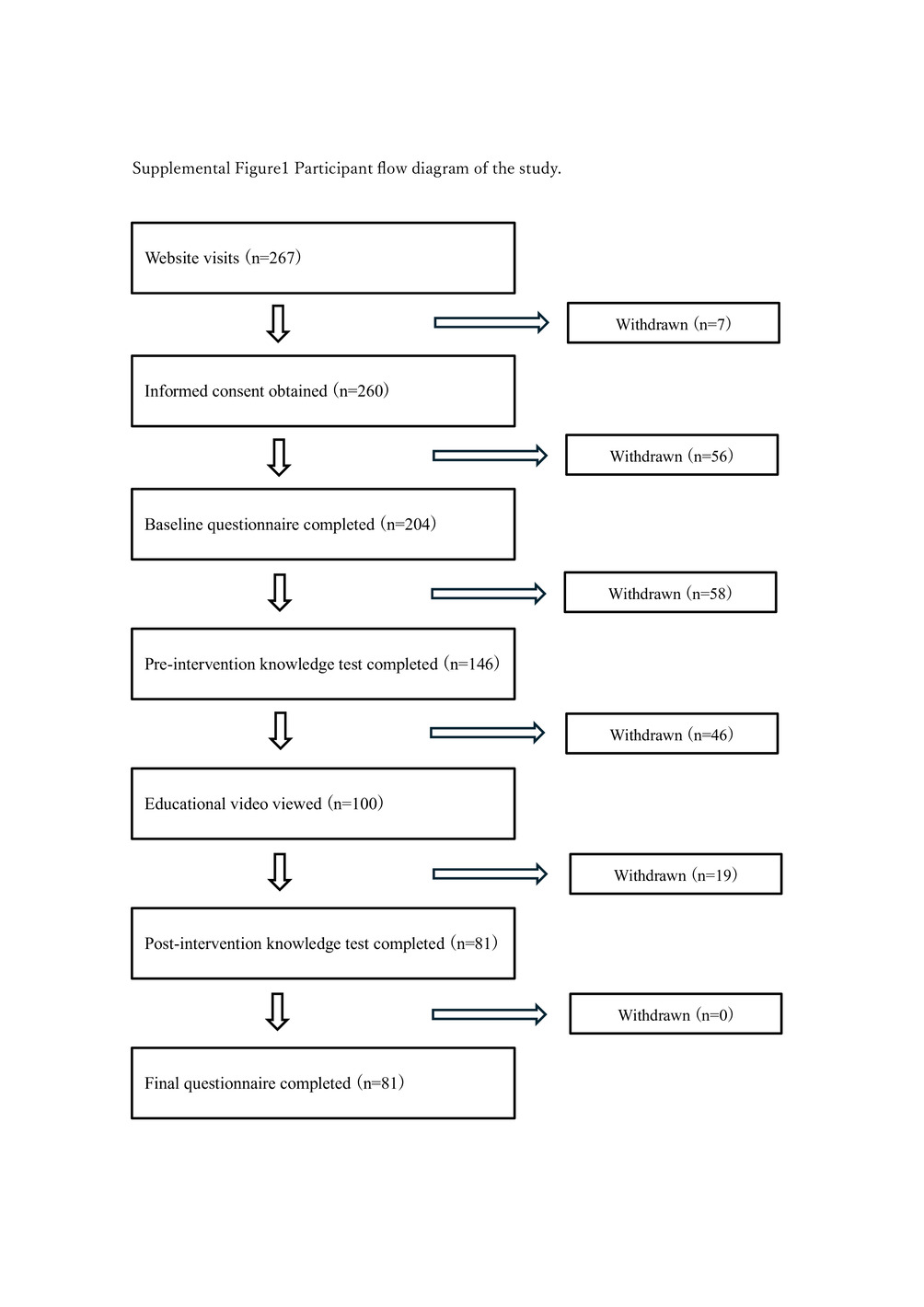

Supplement: Supplementary file 1 [file Image_1.jpeg]
