# Supplementary material for: Short-term effects of video-based education on occupational safety knowledge among commercial divers
Source: Front Public Health. 2026 Apr 15;14:1799866. doi: 10.3389/fpubh.2026.1799866 (PMC13127255; doi:10.3389/fpubh.2026.1799866)
Supplement: Supplementary file 2 [file Table_1.docx]

Supplemental Table 1

| Test Items | correct answer |
| --- | --- |
| **1. Select the one correct statement about semi-saturated tissues.**  　A. A tissue compartment in which half of the inert gas required for full saturation is present. 　B. A tissue compartment in which the inert gas partial pressure equals half of the M-value. 　C. Half-time tissue and semi-saturated tissue refer to the same concept. 　D. I do not know. | A |
| **2. Select the one correct statement about the partial pressure of inert gases.**  　A. The amount of inert gas under the current pressure expressed in pressure units (kPa). 　B. The amount of inert gas under the current pressure expressed as a percentage (%). 　C. The partial pressure of inert gases is the same for helium and nitrogen. 　D. I do not know. | A |
| **3. Select the one correct statement about M-values.**  　A. The maximum total amount of inert gas that a tissue can absorb. B. The maximum total amount of inert gas that a tissue can contain without causing  decompression illness. C. If the value remains within the M-value, decompression illness will not occur. D. I do not know | B |
| **4. Select the one correct statement about the Bühlmann ZH-L16 model.**  　A. A decompression model used to calculate decompression theory. B. It is currently the only decompression model available. C. It can calculate nitrogen but cannot calculate helium. D. I do not know. | A |
| **5. Select the one correct statement about pulmonary oxygen toxicity under the Japanese High-Pressure Work Safety Regulations.**  (UPTD: Unit Pulmonary Toxic Dose; CPTD: Cumulative Pulmonary Toxic Dose)  　A. The maximum allowable value per week under the Japanese High-Pressure Work Safety  Regulations is 2800 CPTD. 　B. One UPTD represents the value obtained by breathing oxygen at 1 atmosphere for 1  minute. 　C. The maximum allowable value per day under the Japanese High-Pressure Work Safety  Regulations is 500 CPTD. 　D. I do not know. | B |
| **6. Select the one correct statement about the gas partial pressure limits under the Japanese High-Pressure Work Safety Regulations.**  　A. The nitrogen partial pressure limit is 400 kPa or less. B. The oxygen partial pressure limit is between 10 kPa and 200 kPa. C. The carbon dioxide partial pressure limit is 10 kPa or less. D. I do not know. | A |
| **7. Select the one correct statement about the M-value safety factor under the Japanese High-Pressure Work Safety Regulations.**  　A. The safety factor is expressed as a percentage (%). B. The safety factor is expressed as a fraction. C. The safety factor is defined according to water depth. D. I do not know. | B |
| **8. Select the one correct statement regarding recommended surface intervals before flying after diving.**  　A. The time before boarding an aircraft is defined in the Japanese High-Pressure Work  Safety Regulations. 　B. Flying is prohibited for 24 hours in all cases. 　C. The recommended waiting time is classified into three categories (≥12 h, ≥18 h, ≥24 h)  depending on the number of dive days and decompression requirements. 　D. I do not know. | C |
| **9. Select the one correct altitude at which diving is generally considered altitude diving.**  　A. 300 m B. 600 m C. 1,000 m D. I do not know. | A |
| **10. Select the one correct statement about the decompression tables specified in the Japanese High-Pressure Work Safety Regulations.**  　A. Decompression tables specified in the Japanese High-Pressure Work Safety Regulations  exist. 　B. The U.S. Navy decompression tables may also be used. 　C. There are no decompression tables, and decompression calculations are performed by the  diver or the employer. 　D. I do not know. | C |
| **11. Select the one correct statement about decompression illness.**  　A. Decompression illness is a collective term that includes arterial gas embolism and  decompression sickness. 　B. Decompression illness and decompression sickness are the same condition with different  names. 　C. If decompression illness occurs, recompression therapy must always be performed. 　D. I do not know. | A |
| **12. Select the one correct statement about arterial gas embolism.**  　A. It is known that breathing gas may enter the arterial circulation due to lung rupture  caused by pulmonary overexpansion. 　B. Arterial gas embolism occurs during descent. 　C. It is generally considered a relatively mild condition. 　D. I do not know. | A |
| **13. Select the one correct statement about decompression sickness.**  　A. It occurs when inert gas dissolved in the body during breathing forms bubbles during  decompression. 　B. Symptoms of decompression sickness appear during descent. 　C. Decompression sickness does not occur if divers follow M-values or diving computer  guidance. 　D. I do not know. | A |
